# A Longitudinal Study of Recurrent Experience of Earthquake and Mental Health Problems Among Chinese Adolescents

**DOI:** 10.3389/fpsyg.2018.01259

**Published:** 2018-07-20

**Authors:** Fulei Geng, Ya Zhou, Yingxin Liang, Fang Fan

**Affiliations:** ^1^School of Psychology, South China Normal University, Guangzhou, China; ^2^Center for Studies of Psychological Application, South China Normal University, Guangzhou, China; ^3^Key Laboratory of Mental Health and Cognitive Science of Guangdong Province, South China Normal University, Guangzhou, China

**Keywords:** disasters, adolescence, acute stress, stress sensitization, longitudinal studies

## Abstract

**Background:** The effects of recurrent exposure to disasters on adolescents’ mental health have rarely been studied. We examined the effects of two earthquake experiences 5 years apart in a longitudinal cohort of Chinese adolescents.

**Methods:** A total of 858 adolescents were assessed in September, 2011 (3.5 years after the Wenchuan earthquake, 1.5 years before Ya’an earthquake, T1) and April, 2013 (1 week after the Ya’an earthquake, T2). Participants’ Wenchuan earthquake experiences and symptoms of post-traumatic stress disorder (PTSD) and depression were assessed at T1, and their Ya’an earthquake experiences, Acute Stress Disorder (ASD), PTSD, and depression were assessed at T2.

**Results:** Structural equation models showed a positive relationship between Ya’an earthquake experiences and symptoms of ASD, PTSD, and depression at T2. T1 PTSD symptoms significantly increased T2 ASD, PTSD, and depression symptoms, and also mediated the relationships between Wenchuan earthquake experiences and T2 ASD, PTSD, and depression symptoms. T1 Depression symptoms also significantly increased T2 ASD, PTSD, and depression symptoms, but only mediated the association between Wenchuan earthquake experiences and T2 depression symptoms. In addition, Wenchuan earthquake experiences moderated the effects of Ya’an earthquake experiences on ASD symptoms and PTSD symptoms but not depression.

**Conclusion:** Repeated to exposure to disasters have adverse additive effects on adolescents’ mental health. Adolescents who experience one disaster may be sensitive to the negative impact of subsequent ones.

## Introduction

At 08:02 Beijing Time (00:02UTC) on April 20, 2013, an earthquake with a magnitude of 7.0 on the Richter scale struck the Ya’an County, Sichuan Province, China, the same region devastated by the Wenchuan earthquake on May 12, 2008. The Ya’an earthquake resulted in 196 people dead, 24 missing, at least 11,826 injured with more than 968 seriously injured. A large number of houses and buildings were destroyed, and the estimated direct economic loss was over 85 billion RMB. About 1.5 years before the Ya’an earthquake, a cohort of senior high school students was surveyed to assess long-term psychological sequelae of the Wenchuan earthquake. The occurrence of the Ya’an earthquake thus provided a unique opportunity to examine effects of repeated exposure to disasters on adolescents’ mental status.

Major disasters such as earthquake could cause myriad mental health problems (Norris et al., 2002), especially post-traumatic stress disorder (PTSD) and depression (Furr et al., 2010; Wang et al., 2013). Retrospective and pre- and post-design studies have suggested that predisaster stressors, predisaster psychopathology, and the severity of disaster exposure are important risk factors for post-disaster mental health problems (Silver et al., 2002; Papanikolaou et al., 2011; Tracy et al., 2011; Fergusson et al., 2014; Caramanica et al., 2015; Bromet et al., 2017). In addition, predisaster psychopathology may play an important mediating role between predisaster stressors and post-disaster psychological distress. In China, there have been not too many studies on post-trauma psychopathology before 2008. Ever since the 2008 Wenchuan earthquake, studies of this sort are accumulating (Zhou et al., 2018). Following the 2013 Ya’an earthquake, some studies have also examined prevalence of mental health problems such as Acute Stress Disorder (ASD), PTSD and depression among children and adolescents affected by this earthquake (Zhang et al., 2015, 2016; Zhou et al., 2016). For instance, in a sample of 2,299 child and adolescent survivors, Zhang et al. (2015) reported the prevalence rates of probable PTSD were 37.4 and 24.2% 3 and 6 months following the earthquake, respectively. However, these studies have not taken the Wenchuan earthquake exposure and related psychiatric symptoms into account. Since the Wenchuan earthquake and Ya’an earthquake affected the same areas, research into the relationship between previous exposures to Wenchuan earthquake and psychological responses to Ya’an earthquake could advance our knowledge on psychological outcomes of recurrent trauma exposure among Chinese samples.

Two possible response patterns have been documented among individuals repeated exposure to disasters: sensitization (Solomon et al., 1987; Robinson et al., 1994; Palgi et al., 2014; Shrira et al., 2014; Garfin et al., 2015) and habituation (Norris and Murrell, 1988; Bleich et al., 2003; Palgi et al., 2015). The stress sensitization refers to the situation that prior trauma heighten a person’s sensitivity to negative outcomes following the subsequent stress (Post, 1992). One recent study examined the relationship between acute stress response to the 2013 Boston Marathon bombings and prior direct and indirect media-based exposure to three collective traumatic events: the September 11, 2001 (9/11) terrorist attacks, Superstorm Sandy, and the Sandy Hook Elementary School shooting. The results indicated that people who experience multiple community-based traumas may be sensitized to the negative impact of subsequent events (Garfin et al., 2015). In contrast, the habituation hypothesis posits that previous trauma exposure may fortify a person for future traumatic or stressful events and reduce the risk of mental health problems in later life. Studies of older adults exposed to several serious floods in Kentucky and Israeli residents affected by ongoing terrorism reported minimal psychopathology despite recurrent trauma exposure and provided support for the habituation hypothesis (Norris and Murrell, 1988; Bleich et al., 2003). Participants’ characteristics (e.g., age) (Breslau et al., 2008; Shrira et al., 2014) and similarity of trauma (Dougall et al., 2000) may contribute to the different responses. Adolescence is a crucial and rapid phase of biological, neurological, and psychological development. This may cause adolescents more vulnerable and having more complex response patterns in face of recurrent traumatic events than adults. To our knowledge, no research has examined these two hypotheses among children and adolescents.

Using a longitudinal sample of adolescents, we examined psychological reactions to Ya’an earthquake on adolescents exposed to Wenchuan earthquake. Based on previous studies (Silver et al., 2002; Papanikolaou et al., 2011; Fergusson et al., 2014; Caramanica et al., 2015; Bromet et al., 2017), we hypothesized that (1) Wenchuan earthquake experiences, Wenchuan earthquake related psychiatric symptoms, and Ya’an earthquake experiences positively predicted psychological distress to Ya’an earthquake; (2) the effect of Wenchuan earthquake on psychological distress to Ya’an earthquake mediated via Wenchuan earthquake related psychiatric symptoms. Given that participants were adolescents exposed to the deadly Wenchuan earthquake, we also hypothesized that that stress sensitization would be most likely to occur. That is the interaction term of Wenchuan and Ya’an earthquake experiences significantly predicted psychological distress to Ya’an earthquake.

## Materials and Methods

### Setting and Participants

**FIGURE 1:**
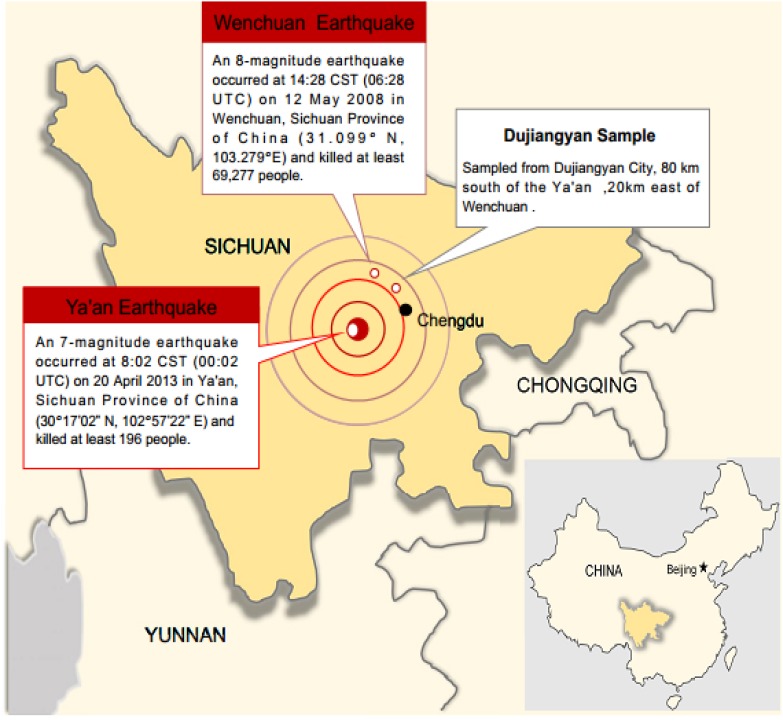
Geographical location of Dujiangyan in relation to the epicentre of the 2013 Ya’an earthquake, China.

Participants were 858 students (318 male and 540 female) recruited from one high school in Dujiangyan, a city affected by both Wenchuan earthquake and Ya’an earthquake. The geographical location of Dujiangyan in relation to the two earthquake epicenters is depicted in **Figure 1**. In Dujiangyan, the Wenchuan earthquake resulted in 3,069 deaths, 4,388 injuries, and 429 missing. A large number of adolescents were directly exposed to the disaster. In the Ya’an earthquake, only one people was reported dead in Dujiangyan, yet it was strongly felt in this area. When the Ya’an earthquake happened, students of the participating high school were having classes and were evacuated urgently to school playground. The evacuation was rather chaotic and many students showed strong stress reactions.

In September, 2011 (T1), 3.5 years following the Wenchuan earthquake and 1.5 years before the Ya’an earthquake, a sample of 1,036 students were initially measured to assess the long-term mental health outcomes of the Wenchuan earthquake. All the students in grade 10th were sampled and there was no exclusion criteria. In April, 2013 (T2), about 1 week following the Ya’an earthquake, a total of 858 students participated in the follow-up survey. In this study, data of participants who completed both surveys was reported. There was no statistical differences in death, missing or injury of family members (χ^2^ = 5.10, *p* = 0.28), house damage (χ^2^ = 3.18, *p* = 0.53), property loss other than house (χ^2^ = 4.91, *p* = 0.30), witness or hearing of tragic scenes (χ^2^ = 4.09, *p* = 0.39), PTSD symptoms (*t* = -1.36, *p* = 0.17) and depressive symptoms (*t* = -1.85, *p* = 0.06) at T1 between participants who completed both surveys and those who not. However, females were more likely to drop out than males (χ^2^ = 8.58, *p* < 0.01).

The Human Research Ethics Committee of South China Normal University approved the study. Written informed consents were obtained from adolescents and their parents before each survey. Participants completed a battery of self-administrated paper-pencil questionnaires in classroom settings during normal school time, under the supervision of psychological professionals from South China Normal University who were experienced in administering psychological and psychiatric assessments.

### Assessments

#### Wenchuan Earthquake Experiences

The participants’ Wenchuan earthquake experiences were measured at T1 using four items: death, injury and/or missing of family members (5 = death of a family member, 4 = missing of a family member, 3 = severe injury of a family member, 2 = moderate injury of a family member, and 1 = none of the above); house damage (5 = completely damaged, 4 = severely damaged, 3 = moderately damaged, 2 = mildly damaged, 1 = not damaged); property loss (5 = entirely lost, 4 = severely lost, 3 = moderately lost, 2 = mildly lost, 1 = not lost); and witness or hearing of tragic scenes (5 = witness a lot, 4 = witness some, 3 = hear a lot, 2 = hear some, 1 = none of the above). The internal consistency of four items measured by Cronbach alpha was 0.65.

#### Ya’an Earthquake Experiences

The participants’ Ya’an earthquake experiences were measured by four items at T2: (1) How chaotic did you feel about the evacuation when the earthquake happened? “1 = very much, 2 = moderately, 3 = slightly, 4 = not at all”; (2) How panicky or scared did you feel when the earthquake happened or during the evacuation? “1 = very much, 2 = moderately, 3 = slightly, 4 = not at all”; (3) In your opinion, how panicky or scared did your peers feel when the earthquake happened or during the evacuation? “1 = very much, 2 = moderately, 3 = slightly, 4 = not at all”; (4) How often were you worried about the occurrence of another earthquake? “1 = always, 2 = often, 3 = occasionally, 4 = never.” The internal consistency of four items measured by Cronbach alpha was 0.72.

#### ASD Symptoms

The Stanford Acute Stress Reaction Questionnaire (SASRQ) was used to measure acute stress responses to the Ya’an earthquake at T2 (Cardena et al., 2000). The SASRQ is a 30-item self-report instrument based on clinical diagnostic criteria, which includes five separate ASD symptom clusters: dissociative symptoms (10 items, e.g., “I felt distant from my own emotions”); intrusive symptoms (6 items, e.g., “I had repeated distressing dreams of the event”); avoidant symptoms (6 items, e.g., “I tried to avoid thoughts about the event”); hyperarousal symptoms (6 items, e.g., “I would jump in surprise at the least thing”) and functional impairments (2 items, “The event made it difficult for me to perform work or other things I needed to do” and “The event caused problems in my relationships with other people”). Participants provide a response for each item using a 6-point scale ranging from 0 (not experienced) to 5 (very often experienced). The SASRQ has been reported to have good reliability and validity among Chinese adolescents (Wen et al., 2009). In this study, we calculated an overall SASRQ score and the Cronbach alpha coefficient was 0.96.

#### PTSD Symptoms

The PTSD Self-rating Scale was used to assess PTSD symptoms at T1 and T2 (Liu et al., 1998). The scale comprises 24 items developed on the basis of the diagnostic criteria of PTSD described in Diagnostic and Statistical Manual of Mental Disorders, Fourth Edition (DSM-IV) and the Chinese Classification of Mental Disorders, Second Edition, Revised (CCMD-2-R). Responses were made on a 5-point scale ranging from 1 (not at all) to 5 (extremely) to indicate the extent to which the symptom affected his or her life. Summing up scores on all items generates a total score, indicating overall severity of PTSD symptoms. The scale has demonstrated satisfactory test-retest reliability (2 week *r* = 0.87) and internal consistency (Cronbach alpha 0.92) among Chinese adolescents (Liu et al., 1998). In this study, the scale also demonstrated good internal consistency, with the Cronbach alpha being 0.93 at T1 and 0.95 at T2.

#### Depressive Symptoms

The Depression Self-Rating Scale (DSRS) was used to assess depressive symptoms at T1 and T2 (Birleson, 1981). The DSRS comprises 18 items on a 3-point scale (0 = never, 1 = sometimes, 2 = most of the time), with a higher total score indicating higher level of depressive symptoms. The DSRS has been found to possess adequate psychometric properties in Chinese adolescents (Fan et al., 2011). In this study, Cronbach alpha for the scale was 0.79 at T1 and 0.81 at T2.

### Statistical Analyses

**FIGURE 2:**
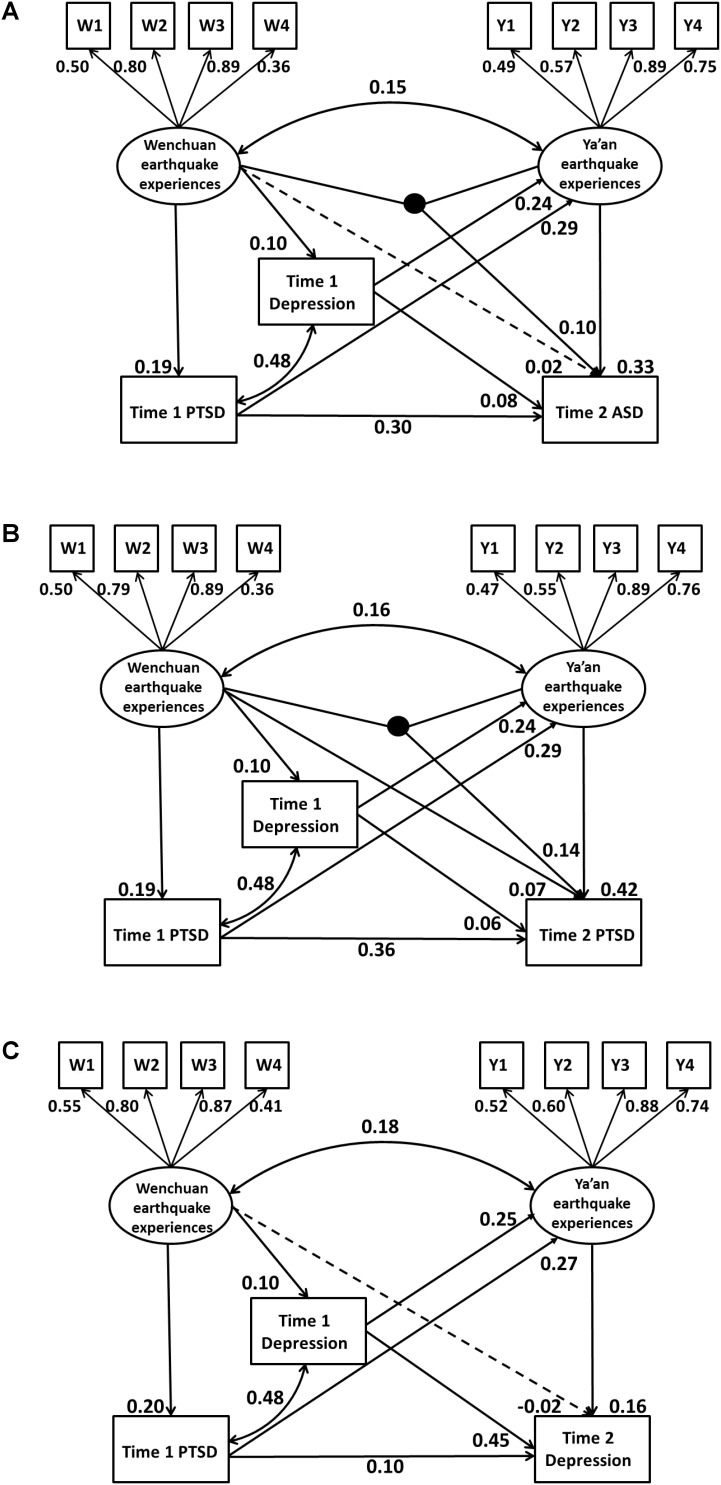
**(A)** The structural equation model of interaction effect of Wenchuan and Ya’an earthquake on ASD symptoms with standardized path coefficients. **(B)** The structural equation model of interaction effect of Wenchuan and Ya’an earthquake on PTSD symptoms with standardized path coefficients. **(C)** The structural equation model of effect of Wenchuan and Ya’an earthquake on depressive symptoms with standardized path coefficients.

In order to examine the study hypotheses, longitudinal structural equation models (SEMs) were performed in Mplus 7.4 (Muthén and Muthén, 2014). According to previous studies (Maslowsky et al., 2015), four analytic steps were conducted. First, a confirmatory factor analysis model was fitted in which Wenchuan earthquake experiences and Ya’an earthquake experiences were modeled as latent variables, each having four categorical indicators. Second, three basic longitudinal SEMs were independently constructed to predict T2 ASD, PTSD and depression symptoms. In these models, Wenchuan earthquake, T1 PTSD and depression symptoms, and Ya’an earthquake experiences were set to directly predict T2 ASD, PTSD, or depression symptoms. To examine the indirect effects of Wenchuan earthquake experiences on T2 ASD, PTSD, and depression symptoms, paths through the mediation of T1 PTSD and depression symptoms were also set. An example of settings of all the path coefficients can be seen in **Figure 2C**. Third, the interaction term of the two latent variables created using the Mplus XWITH function was added into the three basic SEMs. Finally, if the interaction term was significant, Mplus model constraint function was used to compare differences between two sexes in the interaction term.

The models without interaction term were assessed by Chi square (χ^2^), the Comparative Fit Index (CFI), the Tucker-Lewis Index (TLI), and the Root Mean Square Error of Approximation (RMSEA). According to Hu and Bentler (1999), CFI and TLI values above 0.90 and RMSEA values <0.08 represent acceptable model fit. SEMs with and without interaction were compared using a log-likelihood ratio test. A significant χ^2^, generated by comparing -2log-likelihoods indicates that including the interaction term improved model fit. Wald Test was used to examine differences between sexes in the interaction of two latent variables.

## Results

The demographics, Wenchuan earthquake experiences and Ya’an earthquake experiences are presented in **Table 1**. A majority of participants were female (62.9%). The mean ages of participants were 11.4, 15.4, and 17.2 (*SD* = 0.58) years at Wenchuan earthquake, T1 and T2, respectively.

**Table 1:** Demographics and earthquake experiences.

Variables	%
Gender	Male: 37.1; female: 62.9
Age at T1, mean (SD) [range], years	15.40 (0.58) [14–17]
**Wenchan earthquake experiences**	
Death, missing or injury of family members	Death: 13.1; missing: 1.7; severe injuries: 1.7; moderate injuries: 10.8; none of the above: 72.6
House damage	Completely damaged: 17.1; severely damaged: 32.9; moderately damaged: 25.8; mildly damaged: 17.0; Not damaged: 7.2.
Property loss	Entirely lost: 25.4; severely lost: 43.2; moderately lost: 22.6; mildly lost: 7.0; not lost: 1.7.
Witness or hearing of tragic scenes	Witness a lot: 6.8; witness some: 15.5; hear a lot: 31.9; hear some: 23.2; none of the above: 22.6.
**Ya’an earthquake experiences**	
Evacuation in chaos	Very much: 6.2; moderately: 40.0; slightly: 44.6; not at all: 9.2
Panic of peers when evacuating	Very much: 13.8; moderately: 47.7; slightly: 21.5; not at all: 16.9
Scared when the earthquake happened	Very much: 27.7; moderately: 35.4; slightly: 26.2; not at all: 10.8
Worry of occurrence of another earthquake	Always: 29.2; often: 30.8; occasionally; 27.7; never: 12.3


The correlation matrix of study variables are provided in **Table 2**. There were significant correlations among most study variables. The correlations of sex with property loss and witness of tragic scenes during Wenchuan earthquake and chaotic evacuation during Ya’an earthquake were not significant. The correlations of death, missing or injury of family members and witness of tragic scenes during Wenchuan earthquake with T2 depression symptoms were not significant.

**Table 2:** Correlation matrix of study variables.

	1	2	3	4	5	6	7	8	9	10	11	12	13	14
(1) Sex (male = 0, female = 1)	1.00													
**Wenchan earthquake experiences T1**				
(2) Death, missing or injury of family members (W1)	0.07	1.00												
(3) House damage (W2)	0.06	0.41	1.00											
(4) Property loss other than house (W3)	-0.01	0.43	0.71	1.00										
(5) Witness or hearing of tragic scenes (W4)	-0.01	0.31	0.28	0.33	1.00									
**Ya’an earthquake experiences T2**				
(6) Evacuation in chaos (Y1)	0.05	0.16	0.10	0.10	0.10	1.00								
(7) Panic of peers when evacuating (Y2)	0.09	0.15	0.08	0.08	0.16	0.49	1.00							
(8) Scared when the earthquake happened (Y3)	0.24	0.11	0.09	0.08	0.14	0.40	0.51	1.00						
(9) Worry of occurrence of another earthquake (Y4)	0.22	0.10	0.07	0.06	0.07	0.27	0.32	0.69	1.00					
(10) PTSD T1	0.07	0.19	0.13	0.16	0.09	0.09	0.10	0.27	0.32	1.00				
(11) Depression T1	0.14	0.12	0.08	0.07	0.01	0.14	0.18	0.21	0.19	0.49	1.00			
(12) PTSD T2	0.13	0.17	0.11	0.17	0.10	0.24	0.23	0.47	0.52	0.54	0.35	1.00		
(13) Depression T2	0.14	0.04	0.07	0.06	0.00	0.19	0.20	0.20	0.27	0.36	0.53	0.51	1.00	
(14) ASD T2	0.09	0.13	0.07	0.11	0.09	0.28	0.21	0.36	0.43	0.45	0.31	0.70	0.49	1.00


**Correlation coefficient ≥ 0.06, p < 0.05.**

The confirmatory factor model for Wenchuan earthquake and Ya’an earthquake experiences was acceptable, χ^2^ = 131.15, *df* = 19, CFI = 0.97, TLI = 0.95, RMSEA (95% CI) = 0.08 (0.07–0.09). The factor loadings were above 0.5, except for witness of tragic scenes for Wenchuan earthquake experiences, and the two latent variables were related. The three basic SEMs produced adequate fit to the data: model for T2 ASD symptoms, χ^2^ = 197.03, *df* = 37, RMSEA (95% CI) = 0.07 (0.06–0.08), CFI = 0.96, TLI = 0.94; model for T2 PTSD symptoms, χ^2^ = 211.44, *df* = 37, RMSEA (95% CI) = 0.07 (0.07–0.08), CFI = 0.96, TLI = 0.93; model for T2 depression symptoms, χ^2^ = 183.02, *df* = 37, RMSEA = 0.07 (0.06–0.08), CFI = 0.96, TLI = 0.94. The latent variable interaction models for T2 ASD (χ^2^ = 6.63, *df* = 1, *p* = 0.01) and PTSD symptoms (χ^2^ = 18.10, *df* = 1, *p* < 0.01) fitted the data well, but not the interaction model for T2 depression (χ^2^ = 1.34, *df* = 1, *p* = 0.25). The Wald test of sex differences for T2 ASD (*t* = 1.09, *df* = 1, *p* = 0.30) and PTSD (*t* = 0.01, *df* = 1, *p* = 0.92) was not significant.

The path coefficients of basic models (without interaction) for T2 ASD and PTSD are summarized as follows. For T2 ASD: T1 PTSD (β = 0.28, *p* < 0.01), depression symptoms (β = 0.06, *p* = 0.02), and Ya’an earthquake experiences (β = 0.30, *p* < 0.01) significantly predicted T2 ASD; Wenchuan earthquake experiences significantly predicted T1 PTSD (β = 0.20, *p* < 0.01) and depression (β = 0.10, *p* < 0.01), but not T2 ASD (β = 0.01, *p* = 0.80); T1 PTSD (β = 0.28, *p* < 0.01) and depression (β = 0.25, *p* < 0.01) significantly predicted Ya’an earthquake experiences; Wenchuan earthquake exposure covaried with Ya’an earthquake exposure (β = 0.18, *p* < 0.01), T1 PTSD covaried with T1 depression (β = 0.48, *p* < 0.01). For T2 PTSD: T1 PTSD (β = 0.38, *p* < 0.01) and Ya’an earthquake experiences (β = 0.43, *p* < 0.01) significantly predicted T2 PTSD, but T1 depression did not predict T2 PTSD (β = 0.04, *p* = 0.15); Wenchuan earthquake experiences significantly predicted T1 PTSD (β = 0.20, *p* < 0.01) and depression (β = 0.10, *p* < 0.01), but not T2 PTSD (β = 0.03, *p* = 0.32); T1 PTSD (β = 0.28, *p* < 0.01) and depression (β = 0.25, *p* < 0.01) significantly predicted Ya’an earthquake experiences; Wenchuan earthquake experiences covaried with Ya’an earthquake experiences (β = 0.18, *p* < 0.01), T1 PTSD covaried with T1 depression (β = 0.48, *p* < 0.01).

The path coefficients of final models are presented in **Figures 2A–C**. Concerning the model for T2 ASD symptoms, T1 PTSD (β = 0.30, *p* < 0.01) and depression symptoms (β = 0.08, *p* = 0.02) and Ya’an earthquake experiences (β = 0.33, *p* < 0.01) significantly predicted T2 ASD symptoms. The interaction of Wenchuan earthquake experiences and Ya’an earthquake experiences (β = 0.10, *p* = 0.03) also significantly predicted T2 ASD symptoms (see **Figure 2A**). There was a significant indirect effect of Wenchuan earthquake experiences on T2 ASD symptoms via T1 PTSD symptoms, but not via T1 depression symptoms.

**Figure 2B** presents the final model for T2 PTSD symptoms. All variables significantly predicted T2 PTSD symptoms (β = 0.07, *p* = 0.03 for Wenchuan earthquake experiences; β = 0.06, *p* = 0.04 for T1 depression symptoms; β = 0.36, *p* < 0.01 for T1 PTSD symptoms; β = 0.42, *p* < 0.01 for Ya’an earthquake experiences; and β = 0.14, *p* < 0.01 for the interaction of experiences of the two earthquakes). There was also a significant indirect effect of Wenchuan earthquake experiences on T2 PTSD symptoms via T1 PTSD symptoms, but not via T1 depression symptoms.

The model for T2 depression symptoms without latent variable interaction is shown in **Figure 2C**. As we can see, T1 PTSD (β = 0.10, *p* < 0.01) and depression symptoms (β = 0.45, *p* < 0.01) and Ya’an earthquake experiences (β = 0.16, *p* < 0.01) significantly predicted T2 depressive symptoms. There were significant indirect effects of Wenchuan earthquake experiences on T2 depression symptoms via T1 PTSD and depression symptoms.

## Discussion

This study examined mental health outcomes of recurrent experiences of serious natural disasters among Chinese adolescents. The strengths of this study include the longitudinal design, relatively homogenous sample, assessment of the prior disaster-related mental health problems, and analysis of multiple mental health outcomes including ASD, PTSD and depression symptoms. The results demonstrate that repeated experiences of disasters have adverse additive effects on adolescents’ mental health. The findings also provide empirical support for stress sensitization effects – an interaction between Wenchuan and Ya’an earthquake experiences – in liability to ASD and PTSD.

Dujiangyan is located 80 km south of Ya’an earthquake epicenter. Despite personnel casualty and economic loss were not very high, the earthquake was strongly felt in this area. Thus, we assessed subjective disaster-related experiences among adolescents. The results showed that over 50% of participants felt very much and moderately scared when the earthquake happened and over 85% worried about the occurrence of another earthquake. Prior research indicated that subjective traumatic experiences played important roles in development of PTSD with effect sizes ranging from medium to large (Trickey et al., 2012). In the current sample, we also found relatively large dose effect of adolescents’ subjective Ya’an earthquake exposure on their psychological responses. Given that Dujiangyan is relatively far from Ya’an earthquake epicenter, this implies that post-disaster interventions should be extended to high risk population in less affected areas.

Prior trauma exposure could impact persons’ stress responses and mental adaptation processes to later traumas (Silver et al., 2002; Papanikolaou et al., 2011; Fergusson et al., 2014; Caramanica et al., 2015; Bromet et al., 2017). In the current study, Wenchuan earthquake-related PTSD and depression positively predicted mental health problems following the Ya’an earthquake. Extending previous studies (Silver et al., 2002; Caramanica et al., 2015; Bromet et al., 2017), our findings further demonstrate prior trauma imposed its effects on psychological distresses to later trauma mainly through mediation of prior trauma-related psychiatric symptoms. Specifically, Wenchuan earthquake-related PTSD symptoms mediated the effects of Wenchuan earthquake experiences on subsequent Ya’an earthquake-related ASD, PTSD and depression symptoms; Wenchuan earthquake-related depression symptoms mediated the effect of Wenchuan earthquake experiences on subsequent Ya’an earthquake-related depression symptoms. In line with previous longitudinal studies (Stander et al., 2014), these results highlight the important role of a history of PTSD symptoms in development of post-disaster mental health problems. Overall, these findings suggest that multiple disasters have additive effects on adolescents’ mental health problems.

Consistent with the stress sensitization hypothesis (Solomon et al., 1987; Robinson et al., 1994; Palgi et al., 2014; Shrira et al., 2014; Garfin et al., 2015), we found that adolescents with higher level of Wenchuan earthquake experiences were prone to greater acute stress symptoms and PTSD symptoms after subsequent Ya’an earthquake. It is noteworthy that Wenchuan earthquake-related PTSD and depression symptoms were controlled, which may be important confounding factors impacting both subjective Ya’an earthquake experiences and its related psychological reactions (Breslau et al., 2008). One population-based study of childhood adversity indicated that the stress sensitization processes may be different for females and males, i.e., females appeared to be more easily sensitized and only a few major events could increase their susceptibility for PTSD (McLaughlin et al., 2010). In our study, however, stress sensitization effects for ASD and PTSD were similar between sexes. This may be due to that females and males experienced nearly identical level of earthquake exposure. Unlike previous studies of repeated exposure to flood (Norris and Murrell, 1988), terrorist attack (Bleich et al., 2003), and hurricane in adults (Shrira et al., 2014), our findings did not support the habituation hypothesis among adolescents. Participants of previous studies supporting the habituation effect were adults with rich history of experience to bear on crises (Norris and Murrell, 1988; Bleich et al., 2003; Palgi et al., 2015). When facing a new trauma, they have adequate recourses and adaptive coping abilities to overcome the adversity. In our study, participants were adolescents exposed to one of the most deadly disasters in China history. The unexpected Wenchuan earthquake may cause long-term changes in stress reactivity and brain development among children and adolescents (Luo et al., 2012; Du et al., 2015). This may make them more vulnerable and sensitive to subsequent stressors and traumas.

It is interesting to note that the stress sensitization effects were present for ASD and PTSD but not for depression in this study. This disease–specific relationship may be related to abnormal HPA axis function or changes in specific neural circuitry following disasters (Post, 1992; Nusslock and Miller, 2016). In response to disasters, the cortisol levels among survivors will elevate (Luo et al., 2012), which may increase the risk of stress-related disorder such as ASD and PTSD, but not the risk of affective disorders such as depression. Studies have demonstrated that major stressful events can have both short- and long-term effects on the threat-related neural circuitries (Lui et al., 2009; van Wingen et al., 2011, 2012), while the reward-related neural circuitries may be less affected. Thus, stress-related disorders rather than depression may be easier to be kindled following the subsequent trauma. Another possible explanation is that this study only examined short time psychological reactions to Ya’an earthquake. It is possible that stress sensitization effects will be present for depression in later stage following the Ya’an earthquake.

In this study, the effect size of stress sensitive seemed to be small, with the interaction term contributing to approximately 1–2% of the variance of Ya’an earthquake-related ASD and PTSD symptoms. There are two possible explanations. First, Wenchuan earthquake experiences, Wenchuan earthquake-related mental health problems, and Ya’an earthquake experiences were controlled, each of which independently predicted ASD and PTSD symptoms following the Ya’an earthquake. Second, students in the current study experienced slightly objective exposure to the disaster, which may lessen the possible sensitive effect. It should be note that sensitization effects were particularly large among individuals with high level of prior trauma exposure. These individuals may be high risk population of mental health problems. The little increase symptom would be the last straw that overwhelms the camel.

Several limitations of the current study should be acknowledged. First, the experiences of the two earthquakes was retrospectively reported, this may introduce biases. We eliminated the possible memory cofounding by using relatively objective items to measure the Wenchuan earthquake experiences and assessing the Ya’an earthquake experiences with short time interval following the earthquake. Second, the Ya’an earthquake experiences and Ya’an earthquake related psychiatric symptoms were assessed at the same time, this also may introduce biases. Third, the data of pre-Wenchuan earthquake psychiatric status and childhood adversities were not available, which may impact the participants’ responses to the two earthquakes. Future studies can examine the additive effects of childhood adversities and disaster exposure on mental health. Fourth, this pre- and post-design study only followed up adolescents one time. One study reported that stress sensitization occurred among adult survivors after 1 year but not 4 years following a disaster (Smid et al., 2012). The question of duration of stress sensitization following repeated exposure to disasters among adolescents remains unanswered. Finally, psychological reactions to disaster were measured by self-report questionnaires; collecting objective index such as cortisol levels will illuminate possible neuropsychological mechanism of stress sensitization.

## Author Contributions

FG was involved in data analyses and preparation of the manuscript. FF participated in study design and preparation of the manuscript. YZ and YL commented on and revised the manuscript. All authors read and approved the final manuscript.

## Conflict of Interest Statement

The authors declare that the research was conducted in the absence of any commercial or financial relationships that could be construed as a potential conflict of interest.
